# Influence of clinical and psychological variables upon the oral health-related quality of life in patients with temporomandibular disorders

**DOI:** 10.4317/medoral.21746

**Published:** 2017-10-21

**Authors:** Antonio Blanco-Aguilera, Elena Blanco-Aguilera, Rafael Serrano-del-Rosal, Lourdes Biedma-Velázquez, Alejandro Rodriguez-Torronteras, Rafael Segura-Saint-Gerons, Antonio Blanco-Hungria

**Affiliations:** 1 DDS, PhD; Córdoba-Guadalquivir Healthcare District, Andalusian Healthcare Service. Researcher, “Maimonides Institute for Biomedical Research of Córdoba”, Department of Medicine, University of Córdoba, Spain; 2 DDS. MS student in Oral Medicine, Surgery and Implantology, University of Santiago de Compostela, Spain; 3 PhD in Sociology. Research Scientist, Institute for Advanced Social Studies, Spanish National Research Council (IESA-CSIC); 4 Sociologist. Senior Research Technician, Institute for Advanced Social Studies, Spanish National Research Council (IESA-CSIC); 5 MD, Epidemiologist. Andalusian Healthcare Service. Spain; 6 MD, DDS, PhD. Andalusian Healthcare Service. Assistant Professor, Department of Medical and Surgical Specialties, University of Córdoba, Spain; 7 MD, DDS, PhD; Córdoba-Guadalquivir Healthcare District, Andalusian Healthcare Service. Researcher, “Maimonides Institute for Biomedical Research of Córdoba”, Department of Medicine, University of Córdoba, Spain

## Abstract

**Background:**

To analyze the association between the OHIP-14 and the different subtypes making up the clinical and psychological axis obtained using the RDC/TMD.

**Material and Methods:**

407 patients treated at the TMD unit of the Andalusian Healthcare Service were administered the Spanish version of the Research Diagnostic Criteria for Temporomandibular Disorders questionnaire (RDC/TMD), together with the Oral Health Impact Profile questionnaire (OHIP-14). The degree of association between the patients’ score in the OHIP-14 and the clinical and biopsychosocial variables was analyzed through bivariate and multivariate analyses, specifically through linear regression.

**Results:**

89.4% of the treated patients were women, while 10.6% were men, with an average age of 42.08 ± 14.9 years. The mean score and standard deviation for the OHIP-14 was 20.57 ± 10.73. A significant association (*p*<0.05) was observed with the following variables: Axis I, jaw disability checklist, depression, somatization, perceived pain duration, and pain interference with activities of daily living.

**Conclusions:**

The analysis of the relation between self-perceived health in patients with TMD, as measured by the OHIP-14, showed a R2 of 0.3979, with a higher Beta value for the association between the OHIP and patients with both myofascial pain and arthopathy, jaw disability, depression, a higher pain duration and a higher pain interference with activities of daily living.

** Key words:**Temporomandibular disorders, psychological factors, oral health impact profile, public healthcare, research diagnostic criteria for temporomandibular disorders (RDC/TMD).

## Introduction

The term temporomandibular disorders (TMD) includes a wide range of pathologies, whose signs and symptoms involve the temporomandibular joints, the muscles that control them or both (1). The signs are numerous, but pain in the orofacial region is the main symptom, which may appear associated with sounds and/or limitations, involving a certain degree of dysfunction and disability in these patients.

The etiological theories have gone a long way since when Costen defined these disorders and associated them to occlusal alterations due to a loss of posterior support, especially in women; the gender factor, though, has remained unalterable ever since, with proportions of 5 women for each man in the literature. The multi-causal etiology has prevailed, as reflected by authors like Suvinen *et al.* (2), who defends a model with multiple etiological causes, covering a wide range of causes, such as macro-traumas, micro-traumas (particularly bruxism), skeletal and occlusal alterations, or systemic, hormonal and genetic factors, etc.

This model also includes psychological factors such as stress, anxiety or depression as perpetuating etiological causes, which involve an increase in the head and neck musculature’s activity. The role of sleep disorders, either due to obstructive causes like OSAS or to sleep movement disorders like sleep bruxism, has been revealed in the last years, and they entail an inability to relax the orofacial musculature and the rest of the body.

In order to diagnose patients with temporomandibular disorders, the use of the Research Diagnostic Criteria for Temporomandibular Disorders (RDC/TMD) has been standardized, which are based on a dual axis that makes it possible to obtain clinical sub-types in these patients (muscular pathology, disc displacement and intracapsular alterations), either individually or in groups. The second axis, or biopsychosocial axis, allows using a series of items such as pain intensity, graded chronic pain scale, and psychological variables (depression, anxiety and somatization), providing a comprehensive approach to the possible etiological causes.

Applying and introducing the RDC/TMD criteria, with their high specificity and reliability, allowed us throughout the last two decades to standardize the different clinical subtypes (3), as well as the degree of pain intensity, disability and psychological disorders in patients with TMD (4), which has been very useful to compare samples of patients with similar clinical characteristics but ethnically or culturally diverse, enriching the value and the results of this diagnostic method.

The lack of items reflecting the qualitative perception of these patients’ health was perhaps the main handicap of the RDC/TMD in the beginning; however, this was overcome during the last decade by incorporating questionnaires measuring patients’ self-perceived health, which made up for that lack (5).

The OHIP, developed and validated by Slade and Spencer, has been widely used in all fields of dentistry in its two versions: the extended version, including 49 questions (6), and the simplified version, including 14 questions (7). In Spain, the OHIP was validated by Montero-Martin *et al.* (8) by applying it to a sample of adults, and it proved to be a valid, precise and reliable tool to measure patients’ oral health-related quality of life.

The aim of this paper is, on the one hand, to measure TMD patients’ self-perceived health, to which end the OHIP-14 indicator has been used as it is described in the literature, to later analyze the correlation between this perceived oral health and different variables, both physical and psychological. Thus, the paper intends to empirically assess the relation between self-perceived oral health and the chronification of this pathology in patients treated at the Andalusian Healthcare Service’s primary healthcare services, analyzing which variables have an influence in such perception, and to what extent.

## Material and Methods

In order to carry out the study that we present below, we have analyzed the responses of 415 patients with signs and symptoms of TMD treated in the healthcare district of Córdoba (Spain), which belongs to the public Andalusian Healthcare Service. Before being examined, the patients were informed about the study’s protocols and were asked for express permission to fill out the questionnaire. This questionnaire complied with the norms previously established by the ethics committee of the Reina Sofia Teaching Hospital. Eight patients who refused to undergo the examination or to fill out the questionnaire were excluded from the study. The remaining 407 patients were examined by a specialist with 27 years of experience in TMD, and filled out the questionnaire between January 2011 and November 2012.

It is worth mentioning that the main limitation of our study was the lack of a control group to allow us to compare the variability of results among patients with no pathology. The possible bias in assessment and interpretation was corrected by having coauthors unconnected to the clinical aspects of the examination of patients analyze the data.

The criteria for inclusion were being 18 or over (as the RDC/TMD criteria are not validated in patients under 18) and having reported some of the following signs or symptoms: pain in the jaw or the TMJs; limited or restricted range of motion when opening or closing the mouth, or in lateral excursions; and/or joint sounds, with or without pain.

Apart from the refusal to take part in the study or to sign the informed consent form, the following exclusion criteria were also applied: patients who suffered some kind of systemic rheumatic disease (with the exception of fibromyalgia and rheumatoid arthritis), or neurological or autoimmune diseases; patients who had undergone TMJ surgery or head and neck radiation treatment in the two months prior to the study; patients who had suffered head and neck trauma in the two months prior to the study; pregnant patients; patients treated with narcotic analgesics, muscle relaxants or corticosteroids whose treatment could not be suspended one week prior to the study; patients who had been treated with antidepressants or NSAIDs at least in the three days prior to the study; and drug-dependent patients.

To diagnose TMD we used the RDC/TMD designed by Dworking *et al.* (9), more precisely, the Spanish version that explicitly defines each step of the research, together with the instructions to ensure a standardized approach for the sake of possible research purposes. This international questionnaire has been recently replaced by the new Diagnostic Criteria for Temporomadibular Disorders (DC/TMD) (10), but unfortunately it was still not available at the beginning of the study, being at this moment in phase of translation.

According to the RDC/TMD, a diagnosis for patients with TMD is obtained as a result of assessing the patients on the basis of their clinical history and a physical examination using clinical decision algorithms, with the aim of obtaining a clinical classification. In addition, we also intended to obtain sociodemographic and psychological variables so as to use them in our study.

These two psychological variables have been used: degree of depression and non-specific physical findings or somatization. Both indicators are based on the modified version of the SCL 90 scale (11,12). They include the answers to 31 items incorporated into the Axis II of the RDC/TMD. The resulting indicators are the result of adding the scores in each item, then divided by the total number of variables that make up the indicator (20 items in the case of the depression indicator, and 12 items in the case of the somatization indicator). Thus, two indicators are obtained, with values ranging from 0 (no symptoms) to 4 (extreme symptoms), being possible to have any continuous value within the variable.

For the qualitative analysis of self-perceived oral health in patients with TMD, the Spanish version of the OHIP-14 questionnaire was used. This questionnaire comprises 14 questions ( Table 1), each of them with five response categories corresponding to a 5-point Likert scale, where 0 means “never” having suffered problems or pain, and 4 means that the reported pain has been suffered “very often”. The OHIP-14 results in an indicator obtained directly from the sum of the results of each of its 14 items (OHIP-14 = ∑ v1+ v2 +… + v14). We decide to use the OHIP summary score because, as John et al. said (13), the application of the seven original OHIP domain scores leads to diminished statistical power and claims to measure constructs that are not supported by empirical evidence. Instead, the use of one score of the OHIP is sufficient to characterize the oral health-related quality of life.

**Table 1:**
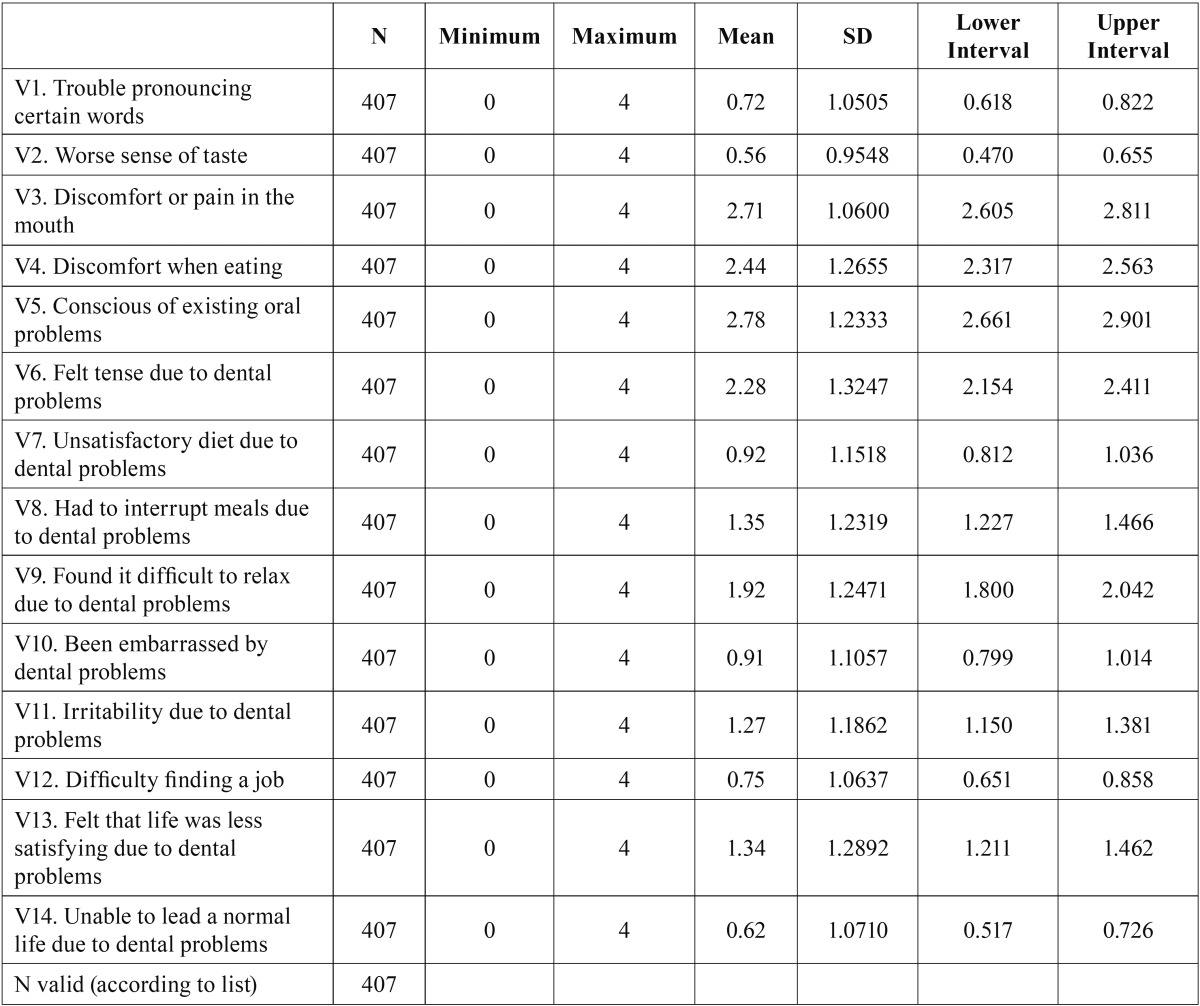
Descriptive statistics of the OHIP-14 items.

The pain interference with activities of daily living measured by the “Graded Chronic Pain Scale” (GPCS) was also calculated, as it is usually described in the literature (9). It was obtained using two indicators: A) “Pain intensity”, determined using visual analogue scales (VAS) (current pain intensity, maximum pain intensity and average pain intensity /3). B) “Degree of disability”, determined by quantifying the number of disability days and the degree of disability obtained from the sum of the VAS scores (how the disability affects patients’ daily, recreational and work activities). The values are quantified from 0 to 4 (0 = No pain; 1 = Low intensity (< 50) and no disability; 2 = High intensity (> 50) and no disability; 3 = High disability and moderate limitation; 4 = High disability and high limitation).

It is also important to note that the pain duration variable was divided into two groups, one made up of patients who reported having suffered from pain for less than a year, and another group made up of patients who reported having had pain for one year or more. These groups were formed empirically on the basis of a hierarchical segmentation analysis where the dependent variable was the OHIP-14 score and the independent variable was pain duration measured in months. This empirical analysis allowed us to statistically discriminate those groups which differed in relation to the dependent variable. More precisely, it provided an appropriate number of groups and established the cutoff point taking into account the statistical differences observed in the empirical evidence.

**Figure 1:**
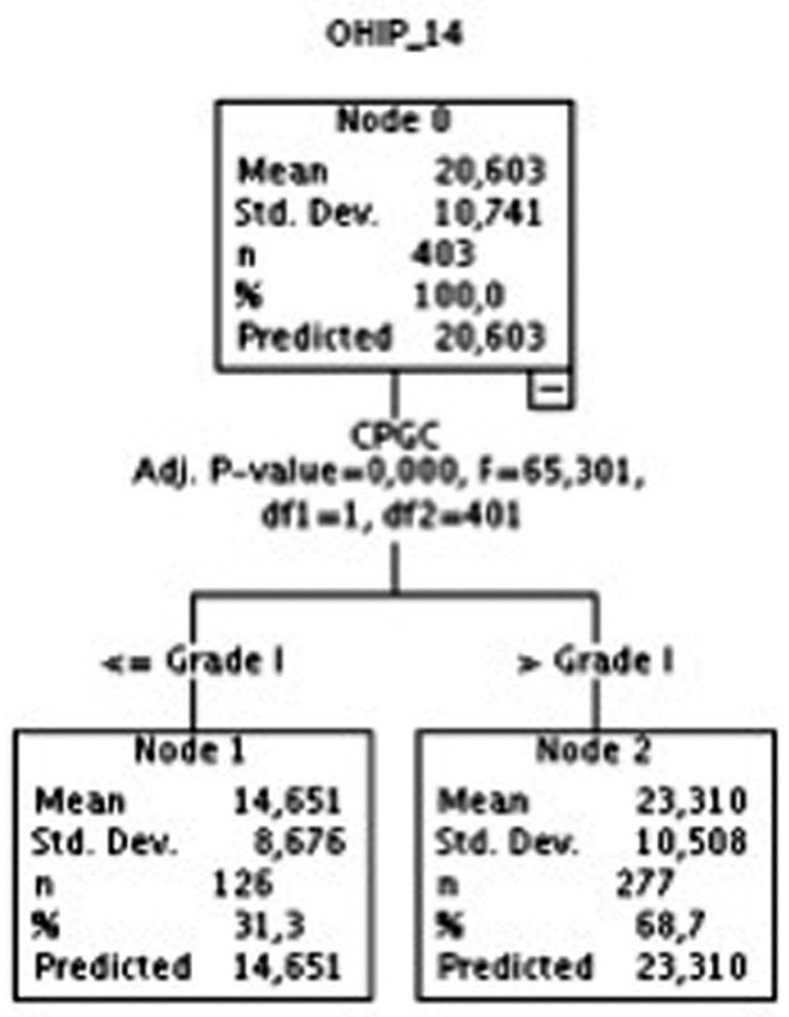
Dichotomization of the CPGS.

**Figure 2:**
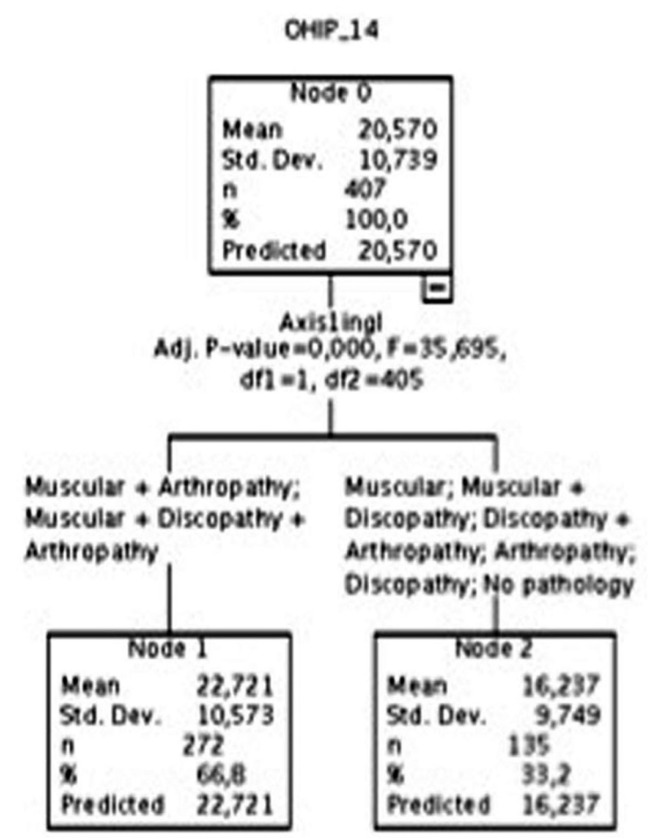
Dichotomization of the Axis I.

As regards the statistical analyses carried out to achieve our objective, we first performed a contrast of means between the OHIP-14 scores and the analyzed variables (using Snedecor’s F-statistic or Spearman’s Rho, depending on the level of measurement of the analyzed variable). After that, a hierarchical segmentation analysis of the variables GPCS and Axis I was performed, with the aim of empirically observing for which categories of such variables their association to the OHIP-14 variable was more significant (Figs. 1,2).

As may be observed in Figure 1, as regards the OHIP-14 variable, two groups of the CPGC variable were created, one including the pain from grade 0 to grade I, and another group including the values higher than grade I (i.e., grades II, III, and IV). As regards Axis I, two groups were also formed in the hierarchical segmentation analysis: one comprising all the people who suffer from a muscular pathology along with an arthropathy, and another group for the rest of categories, as may be observed in Figure 2.

Finally, we performed a linear regression analysis, using the OHIP-14 indicator as the dependent variable and the variables that were found to be statistically significant in the bivariate analysis as explanatory variables.

We tested for collinearity between the independent variables and, in order to avoid it, we analyzed the partial correlations and the indicators of collinearity, thus decreasing the number of variables that were ultimately included in the model. High collinearity was the reason to exclude the somatization variable from the regression model. The data were analyzed using the statistical software package SPSS, version 15.

## Results

The sample was made up of 365 women (89.7%) with a mean age of 42.15 ± 14.63 (mean ± standard deviation), and 42 men (10.3%) with an average age of 41.48 ± 17.28. This representation corresponds to a 8.4 W:M ratio. The age range is from 18 to 83 years.

**Figure 3:**
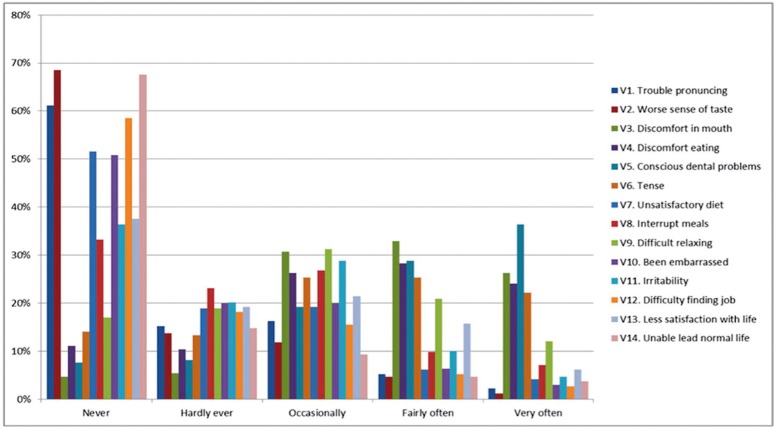
OHIP-14.

The distribution of the mean scores and standard deviations for each indicator are shown in  Table 1 and Figure 3, where the most prevalent mean scores are those related to questions 3, 4, 5 and 6 (discomfort in the mouth, discomfort when eating, existing oral problems, and tension due to such problems); the mean scores for these items were over 2. In contrast, questions 1, 3 and 14 (trouble pronouncing certain words, worsened sense of taste, and incapacity to lead a normal life) reveal average scores that are clearly lower than the rest of variables in the questionnaire, i.e., they are less prevalent in the patients in the study.

Figure 3 shows the distribution of the responses, expressed as frequencies in a bar chart for each of the questions of the OHIP-14 indicator. Here, the modal score is the “never” response, except in the variables directly asking about pain or oral discomfort, where the most frequent response is “fairly often” and “very often”, and “never” and “hardly ever” are very infrequent responses among these patients.

Beginning with the descriptive values of some variables, the score obtained from the mean and the standard deviation of the OHIP-14 was 20.57 ± 10.73 (mean ± standard deviation), thus with a 95% CI (19.52 – 21.61).

The jaw disability variable, in turn, obtains a mean score of 0.33, with scores ranging from a minimum of 0 to a maximum of 1. The pain interference with activities of daily living indicator interpreted as the “degree of disability associated to pain” is made up of each patient’s pain intensity variable, together with the variable measuring the disability generated by that pain in each of the activities the patient performs. The former reveals an average score of 1.78, with a 95% CI range (1.71-1.86), which would mean that 95% of the sample shows pain intensity scores above 50 points in the VAS scale, but they do not report disability in their daily activities.

Depression, measured by a 0-4 scale, shows an average score of 1.33, with a CI range between 1.25-1.41, which may be interpreted as a severe degree of depression, since, according to the RDC/TMD International Consortium, depression is considered severe when the average score is over 1.105.

The average score for the pain duration variable was 1.59, with a CI range between 1.55-1.64. If we take into account that the scores for this variable are: 1, whether the patient has had pain for a period of less than 12 months, and 2, whether the pain duration is more than one year, we note that the average score is slightly over the midpoint, which means that the patients who have been having pain for more than one year slightly outnumber those who have had pain for a shorter period of time.

 Table 2 and  Table 3 show the bivariate analysis between the OHIP-14 and the other variables included in the study. As may be observed, the variable defined as “pain interference with activities of daily living” presents a progressive increase in the mean scores in accordance with the degree to which it is affected. Likewise, once this variable has been dichotomized, it may be observed that the patients in groups II, III and IV, i.e., those with more intense pain or a certain degree of disability, have a statistically worse self-perceived quality of life.

**Table 2:**
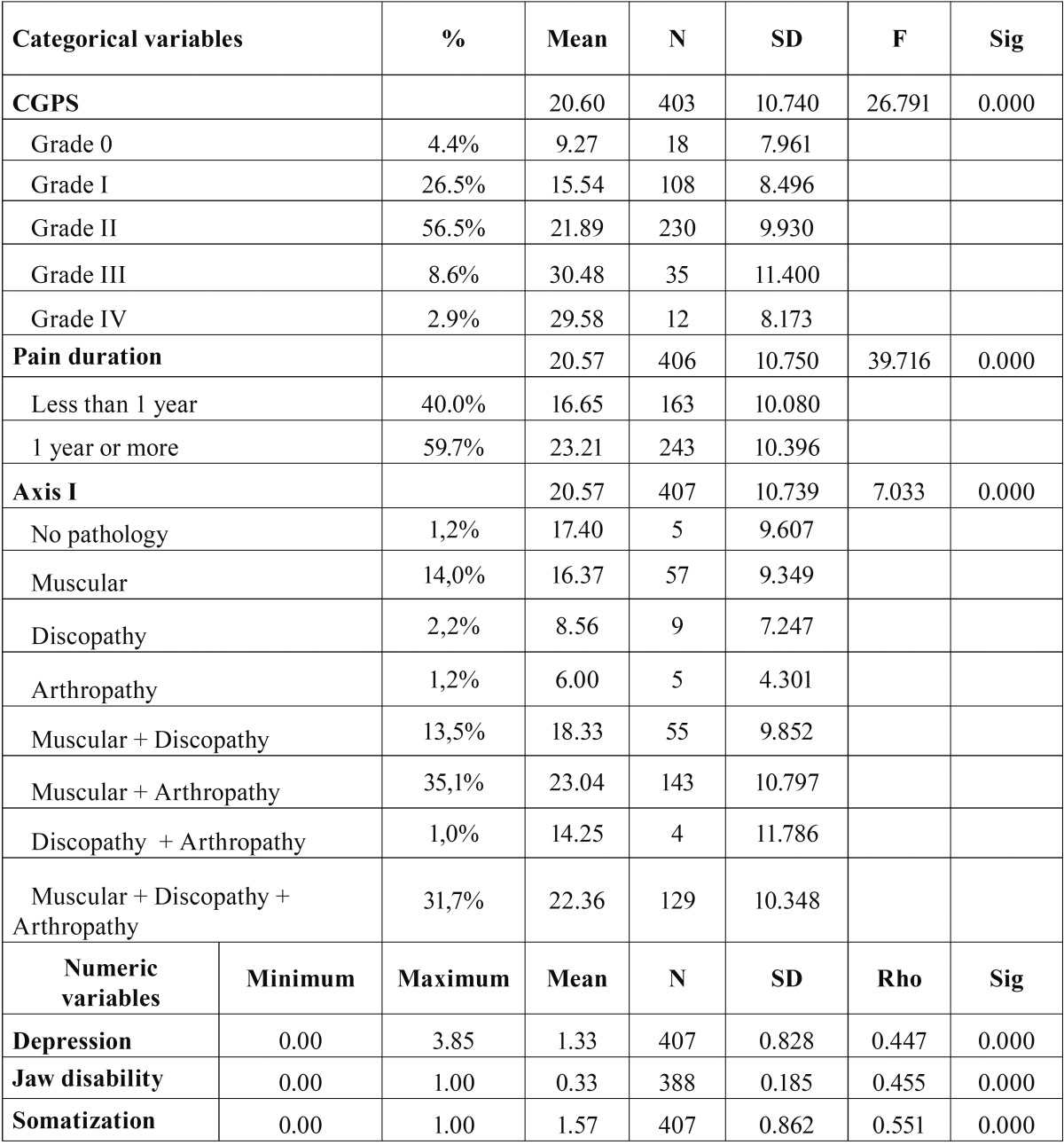
Difference of means with the OHIP-14.

**Table 3:**
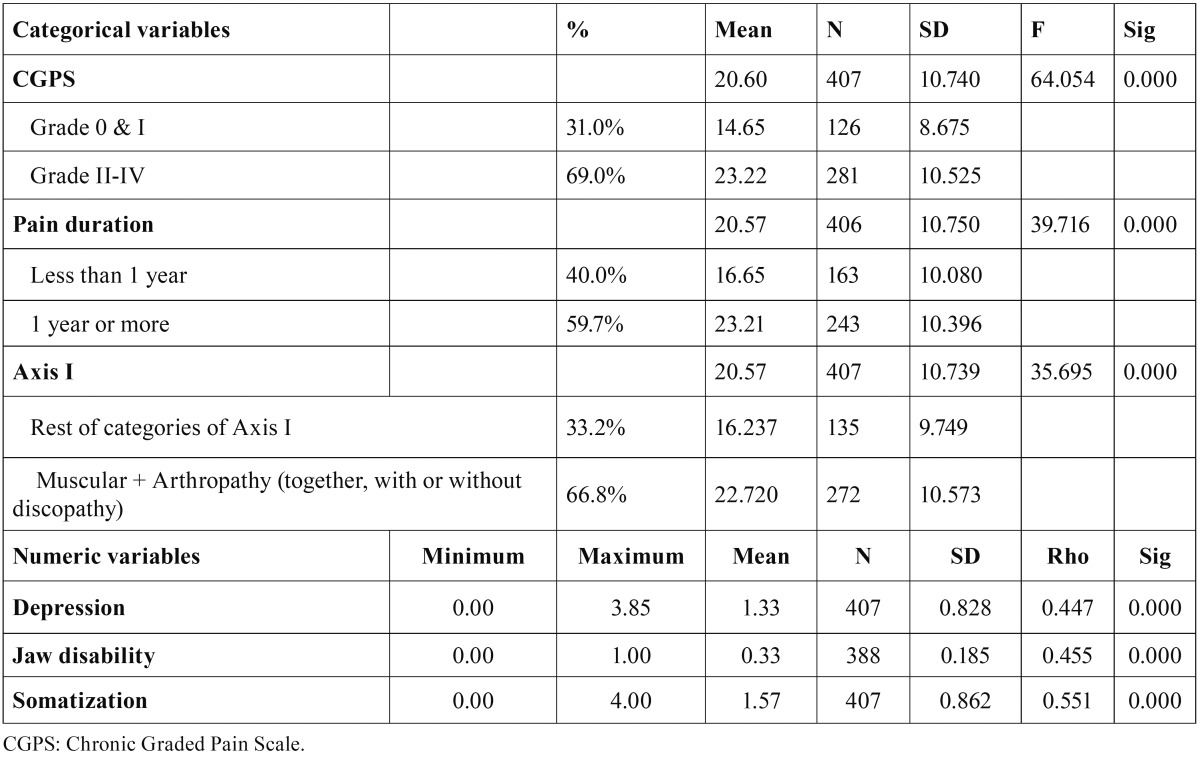
Difference of means with the OHIP-14 after dichotomization.

In the relation between the OHIP-14 variable and pain duration, we have noted that there is a clear difference in the scores of both subgroups, as the patients reporting pain for more than one year obtain a score 6.6 points higher in the OHIP-14 than those who report pain for less than one year.

As regards the relation between the OHIP-14 with the Axis I variable (which includes all the clinical subgroups), it may be observed that the OHIP scores for the muscular subgroups associated to arthralgia and those muscular pathologies accompanied by arthralgia and discopathy are the ones with a worse perception of their oral health, followed, with 6 points less, by the rest of patients.

As far as the Axis II or biopsychosocial axis is concerned, two variables were taken into account: the depression variable and the jaw disability variable, both with a high significance value (*p* ≤ 0.05), as is the case in the rest of the studied variables.

The last table,  Table 4, shows the values obtained from a linear regression analysis using the OHIP-14 as the dependent variable and, as the independent variables, the Axis I clinical variable, pain duration, pain interference with activities of daily living, and the biopsychosocial variables (depression and jaw disability). All the variables are significant in this regression model. Which, overall, is significant and has a high coefficient of determination (R2), which accounts for 39,79% of the OHIP-14 variance.

**Table 4:**
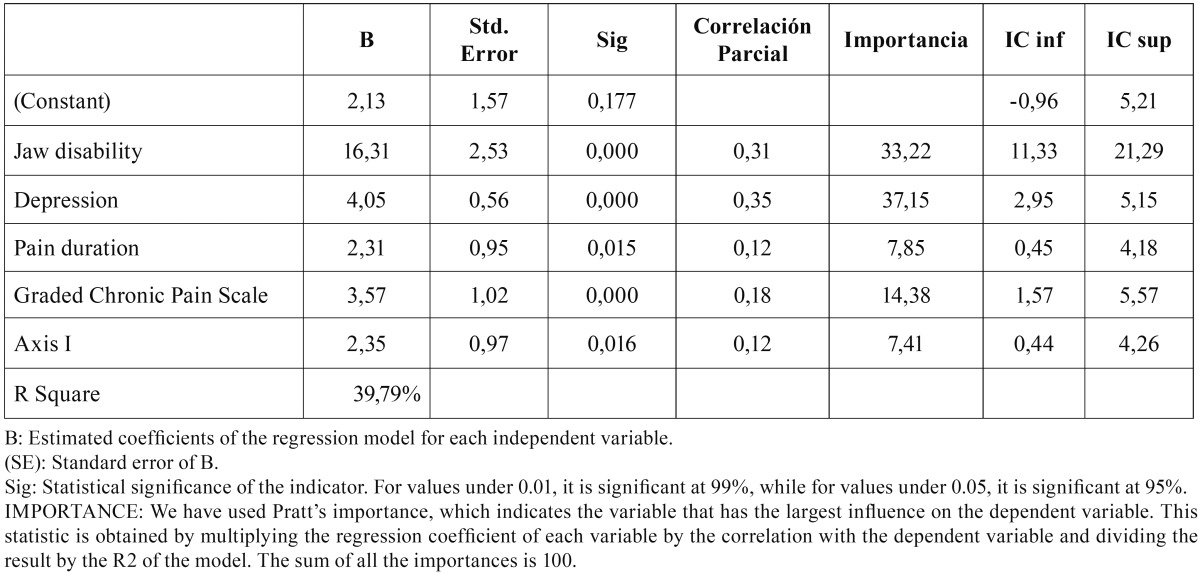
Analysis of the linear regression of the OHIP-14 with the independent variables.

In  Table 4, together with the coefficients of determination and the partial correlation, the value of the Pratt’s Importance statistic is presented, which is interpreted supposing that the coefficients of all variables account for 100% of the model’s R2, and analyzing the “importance” or effect of each of those variables in that variance. This way we are able to easily observe that the variable with the greatest influence, the one with the highest importance in the variance of the OHIP-14 sore, is the depression variable, which would account for almost 37,15% of the model’s R2, followed by the jaw disability variable, which would account for 33,22%, and the graded chronic pain scale, accounting for 14,38%.

It is also important to observe that the Betas in all variables are positive, which means that they have a positive relation with the dependent variable. Thus, the increase in pain duration, chronic pain grade, jaw disability, depression degree or the presence of both myofascial pain and arthropathy entails an increase in the OHIP-14 indicator.

## Discussion

Self-perceived oral health-related quality of life questionnaires have begun to be used in patients with temporomandibular disorders and orofacial pain in the last decade, being developed by Segù *et al.* in the University of Pavia in 2003, using the complete version of the OHIP-49 to that end (5). Later, in 2006, John et al described the validity of the short OHIP questionnaire made up of 14 questions in German patients with temporomandibular disorders and orofacial pain, concluding that its use is perfectly reliable and valid, and recommending it in future longitudinal studies with bigger samples (14).

Later, two other versions of the same questionnaire have been published: a short version with five questions, known as OHIP-5, which, unlike the OHIP-14, has not managed to obtain enough validity to be able to substitute the complete version (15); another version was created to be specifically used with patients with temporomandibular disorders. This OHIP-TMD questionnaire includes 22 questions more focused on this type of pathology but, as stated by its own authors, it should be limited to clinical use in specific TMD patients, rather than in epidemiological studies, since using it instead of OHIP-14 or OHIP-49 might decrease the possibility to compare the possible differences between TMD and other diagnostic entities in their effects on the patient’s quality of life (16).

The validity of the OHIP-14 questionnaire in different versions and languages is clearly accredited in the literature, and it has been validated by different authors. In Spain, Montero *et al.* (17) validated the questionnaire. The study that we have presented in the previous pages describes a mean score of 20.5 points in the OHIP-14, showing a rather compressed confidence interval, with a range of barely 3 points. If we compare this with the results described by Schierz *et al.* (18), ours is 6.5 points higher, as they obtained a mean score in the OHIP-14 of 14 points in patients diagnosed with TMD, a mean score that increases in 8 points in patients reporting dental anxiety, reaching 22.4 points. However, in that same study, the OHIP mean score for the general population was 4.1. Miettinen *et al.* (19) obtained similar results, with an OHIP mean score lower than ours: 15.7 points in patients with this type of pathology.

These differences might be due to the heterogeneity in the distribution of the different TMD subtypes in our sample, with a very high presence of patients with a muscular pathology, probably because the study was carried out at a non-hospital primary care center. While in other studies, like Miettinen *et al’s*. (19), the proportion of patients with some kind of myopathy is around 65%, in our sample that proportion reaches 94.3%. These differences, together with the fact that the patients suffering this type of muscular pathology are precisely the ones with a bigger impact on their quality of life, as reported by some authors (20) and shown by our own results as expressed in  Table 3, could help us explain why we obtained a higher OHIP mean score.

We also deem very important to highlight that in our sample we have noted that the “pain interference with activities of daily living” variable increases its mean score as the degree to which the patient is affected grows. In fact, the most advanced grades (III and IV), which assume that these patients suffer from disability, show a OHIP-14 mean score 20 points higher than that of the patients who do not report pain intensity or disability as a consequence of such pain. These results would be in line with those obtained by Alajbeg v (21), who reported a Fisher’s value for pain intensity and the OHIP even higher than the one in our study. In this case, just like in John *et al’s.* study (22), there is a difference of almost 50 points between grade I and grade IV, although it should be noted that this author used the longer OHIP-49 questionnaire.

The difference of mean scores in the pain duration variable was 6.5, with a Fisher’s value of 39.7. This may be interpreted as most patients having a worse perception of their oral health-related quality of life in accordance with the duration of their painful pathology. This large difference reveals that, as suggested by several authors (23,24), the chronification of pain has an important effect on the deterioration of the patient’s quality of life.

If we analyze the relationship between the OHIP-14 mean scores and each of the combined subgroups comprising the clinical axis or Axis I of the RDC/TMD, we observe a clear preponderance of patients with myofascial pain associated to other subtypes, while the patients with discopathies or arthropathies with no muscular involvement have a better perception of their oral health. Just like in our case, other authors like Barros (25) observe an association between a drop in patients’ self-perceived oral health and certain Axis I subgroups, such as muscular pathology and arthralgia, and no association in patients with discopathy. The conclusions of this study are particularly relevant, taking into account that the subgroups associated to a worse self-perceived oral health are precisely those that usually report more pain intensity (muscular and articular pathologies), as opposed to discopathy, which may be painless (26). This points to the important association between pain intensity and a worse self-perception of patients’ oral health-related quality of life (27).

Likewise, a worse self-perception of their oral health has been widely reported in patients with TMD who also have a psychosomatic pathology like depression (14), with highly significant values in this relationship. This may also be clearly noted in our study, specifically in those patients who have a higher grade of depression and more jaw disability, which occurs sometimes. This characteristic, having a higher grade of depression, is precisely the one with the greatest influence in our analysis when it comes to accounting for the variation in the OHIP-14 scores. In other words, the psychosocial aspects, as pointed out by other studies (19,24,28), are inevitably essential to analyze orofacial pain, as pain, far from being a strictly biological element, has a psychological and social component, as revealed by many studies both in the field of medical research and in the field of social research (29,30). This is extremely important, as the patients’ biological, psychological, and social characteristics are elements that cannot be dissociated when it comes to explaining any phenomenon which, like pain, has a complex nature, and whose social side has previously been documented by the authors (31).

## Conclusions

Looking at these results, we can conclude that those patients with more pain intensity, linked to a higher disability to develop their professional and personal lives, have a worse perception of their oral health, which even worsens in accordance with the duration of the pathology.

We would also like to highlight that, within the different categories the temporomandibular disorders are broken down into, those patients with a muscular pathology accompanied by arthralgia perceive their oral health-related quality of life significantly worse than the rest of patients, which may be accounted for by the fact that these patients are the ones with the highest pain intensity. Regarding the psychological variables, both depression and jaw disability seem to be associated with a worsening in the oral health related quality of life.
